# Real-time programmable metasurface for terahertz multifunctional wave front engineering

**DOI:** 10.1038/s41377-023-01228-w

**Published:** 2023-08-07

**Authors:** Feng Lan, Luyang Wang, Hongxin Zeng, Shixiong Liang, Tianyang Song, Wenxin Liu, Pinaki Mazumder, Ziqiang Yang, Yaxin Zhang, Daniel M. Mittleman

**Affiliations:** 1 Sichuan THz Communication Technology Engineering Research Center, School of Electronic Science and Engineering, University of Electronic Science and Technology of China, Chengdu, 611731 China; 2 Yangtze Delta Region Institute (Huzhou), University of Electronic Science and Technology of China, Huzhou, 313000 China; 3 Zhangjiang Laboratory, Shanghai, 201204 China; 4 National Key Laboratory of Solid-State Microwave Devices and Circuits, Hebei Semiconductor Research Institute, Shijiazhuang, 050051 China; 5 Aerospace Information Research Institute, Chinese Academy of Sciences, Beijing, 100094 China; 6 University of Chinese Academy of Sciences, School of Electronic, Electrical and Communication Engineering, Beijing, 101408 China; 7 Department of Electrical Engineering and Computer Science, University of Michigan, Ann Arbor, MI 48109 USA; 8 School of Engineering, Brown University, Providence, RI 02912 USA

**Keywords:** Terahertz optics, Metamaterials

## Abstract

Terahertz (THz) technologies have become a focus of research in recent years due to their prominent role in envisioned future communication and sensing systems. One of the key challenges facing the field is the need for tools to enable agile engineering of THz wave fronts. Here, we describe a reconfigurable metasurface based on GaN technology with an array-of-subarrays architecture. This subwavelength-spaced array, under the control of a 1-bit digital coding sequence, can switch between an enormous range of possible configurations, providing facile access to nearly arbitrary wave front control for signals near 0.34 THz. We demonstrate wide-angle beam scanning with 1° of angular precision over 70 GHz of bandwidth, as well as the generation of multi-beam and diffuse wave fronts, with a switching speed up to 100 MHz. This device, offering the ability to rapidly reconfigure a propagating wave front for beam-forming or diffusively scattered wide-angle coverage of a scene, will open new realms of possibilities in sensing, imaging, and networking.

## Abstract (web summary)

A programmable metasurface based on AlGaN/GaN HEMTs is demonstrated for terahertz multifunctional wavefront engineering in real time.

## Graphical abstract

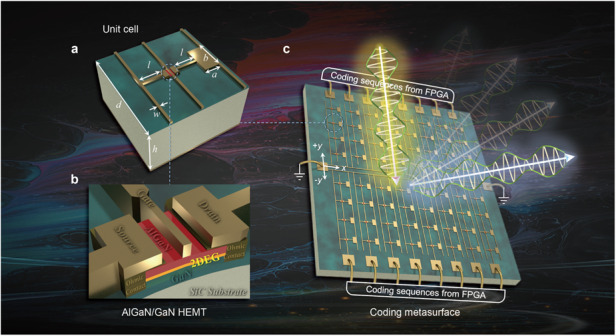

## Introduction

In recent years, the THz band (0.1–10 THz) has become a focus of worldwide research for the sixth-generation (6 G) wireless communications, radar detection, spectroscopic imaging, biomedical sensing^1–7^, and other promising fields. For all of these applications, systems will require methods for agile manipulation of THz wave fronts. A broad set of wave front engineering capabilities will be required to meet the demands for creating non-line-of-sight links, directional signal enhancement, interference suppression, and computational imaging^8–11^. Existing strategies rely on mechanical motion, which is far too slow for most requirements^12^, or on phased array systems, which suffer from considerable system complexity and fabrication challenges, as well as high power consumption^13^. A more general approach that overcomes these difficulties is urgently needed.

One promising strategy for THz wave front engineering that has attracted a great deal of attention recently is that of active metasurfaces. These devices, consisting of a dense subwavelength array of actively switchable elements, can in principle offer enormous flexibility for dynamic control of wave fronts, with low power consumption and high scalability. At THz frequencies, the method of fabricating the tunable components is much more challenging than at lower frequencies; this determines the capabilities of the resulting device. Researchers have explored various types of materials (e.g., liquid crystals^14,15^, semiconductors^16–21^, graphene^22^, and phase-change materials^23–27^) as tunable components, instead of conventional diode structures used typically in the microwave band. Among these tunable materials, electrically modulated ones are more beneficial to addressable and scalable controls in all-electron systems. For example, a liquid-crystal-based metasurface has recently achieved basic 1-bit programmable beam steering in both reflective^14^ and transmissive^15^ modes. Besides, the complementary metal-oxide-semiconductor (CMOS)-based metasurfaces successfully reach the highest response speed up to 5 GHz^17^, and the highest beam-scanning precision of 1° by integrating phase shifters in a half-wavelength element^18^, which however demands customized fabrication processes with high costs. These related works in recent years are listed and compared in Table 1, along with some critical features for THz beam control including element size, response speed, beam coverage, and scanning precision. A THz metasurface that balances the performances of response speed and beam-scanning precision and enables fully versatile wave front engineering has yet to be demonstrated, due to limitations of both the switching mechanisms and the methods of active tuning.

**Table 1:** Comparison of the related metasurfaces for THz beam steering

References	Control Way/Mode	Frequency (THz)	Element number	Element size	Response speed	Measured beam angles	Beam-scanning precision	Other demonstrated functions
Ref. ^14^ (2020)	Liquid crystal /Reflection	0.672	24 × 50	0.38λ	100 Hz	8.5°, 13.5°, 31.5°	N/A	N/A
Ref. ^15^ (2021)	Liquid crystal /Transmission	0.462	48 × 48	0.49λ	1 kHz	−9°, −15°, −29° 9°, 16°, 30°	N/A	N/A
Ref. ^27^ (2022)	Vanadium dioxide /Reflection	0.425	48 × 90	0.23λ	0.38 Hz	0°, 13°, 18°, 22°, 29°, 43°	N/A	N/A
Ref. ^48^ (2019)	HEMT-CMOS /Reflection	0.235	32 × 32	0.55λ	500 MHz	−40°, −20°, 0°, 20°, 40°	N/A	N/A
Ref. ^17^ (2020)	65nm-CMOS /Transmission	0.3	24 × 24	0.14λ	5 GHz	−30°, 0°, 30°	N/A	Holographic imaging
Ref. ^18^ (2022)	25nm-CMOS /Reflection	0.265	98 × 98	0.5λ	100 kHz	±60°	1°	Scanning imaging
**This work**	**GaN HEMT** **/Reflection**	**0.34**	**64** × **64**	**0.23λ**	**100** **MHz**	**20°–60°**	**1°**	**Multi-beam steering** **Diffuse scattering** **Tracking transmission**

The bold entries highlightthis article’s work in comparison with other works

Here, we demonstrate a metasurface which can satisfy the needs of multiple diverse system requirements for wave front manipulation, along with a comprehensive consideration regarding response speed and beam-scanning precision. This work builds on our earlier efforts which employed GaN/AlGaN high electron mobility transistor (HEMT) as the active switching element. These HEMTs offer numerous advantages, including a sizeable dynamic carrier density range, a high electron drift velocity, a smaller parasitic capacitance, and lower power dissipation. Earlier work with these GaN HEMTs has achieved nanosecond-level response speed and large phase shifts in the THz regime^28–30^. In this study, we design an asymmetric resonant structure which incorporates a HEMT as the individual meta-element in our array. This novel design overcomes the limitations caused by parasitic capacitance in many previous designs, and enables close (sub-wavelength) spacing of the individual elements without the need for integrated amplification or phase-control circuitry. These switchable elements are then electrically connected to each other in groups to form an array of sub-arrays architecture. The sub-arrays are distributed symmetrically on either side of a central ground line, a layout that offers significant improvements in the versatility of the structure. The resulting metasurface can be operated as a real-time 1-bit active metasurface. Using this device, we demonstrate quasi-continuous beam scanning, multi-beam steering, and even the generation of diffuse wave fronts as required for some computational imaging applications^31–33^. We also demonstrate real-time beam tracking by transmitting a point-to-point single-tone signal to a moving receiver, as would be required in a mobile THz network. These demonstrations illustrate how this device can fill the need for multifunctional THz wave front engineering.

## Results

### Design of the meta-element and coding metasurface

**Fig. 1:**
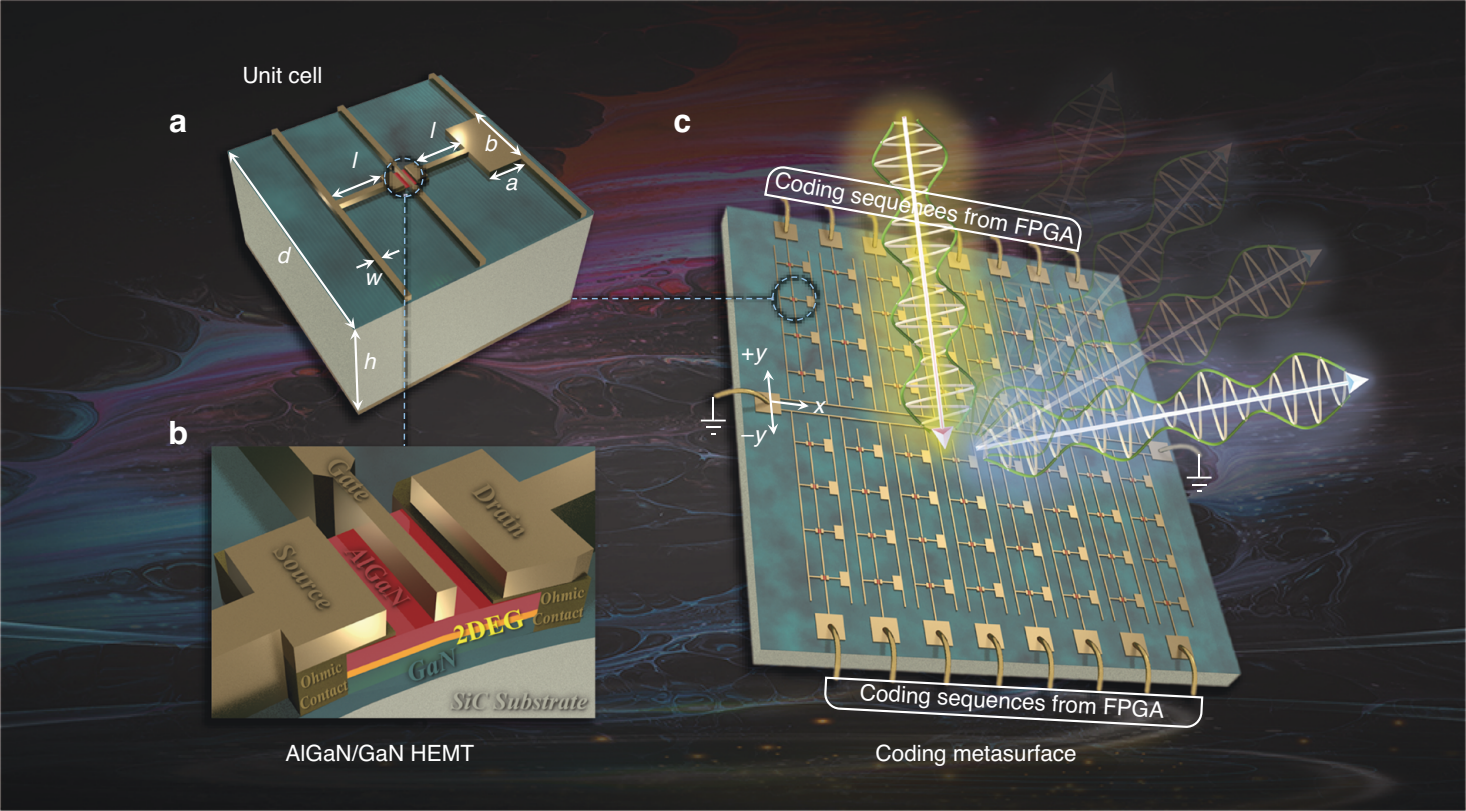
3D schematic diagrams of the designed meta-element and coding metasurface. **a** The 2DEG-embedded meta-element, where the labeled geometric dimensions are: element period *d*, SiC thickness *h*, strip length *l*, and width of strips and bias-ground wires *w*. The patch (width *a*, length *b*) on one side breaks the symmetry of the meta-element with respect to the central gate electrode. **b** The cross-sectional diagram of the AlGaN/GaN HEMT. **c** The real-time controlled coding metasurface. As one of the functions, the sketch of beam scanning carried with modulated information is shown on the metasurface

The schematic diagrams of the hybrid meta-element and coding array are shown in Fig. 1. As shown in Fig. 1a, b, the meta-element is composed of a two-dimensional electron gas (2DEG) (shown in yellow) embedded in a gold pattern. This constitutes the core resonant element. These elements are fabricated on a SiC substrate covered by a nanoscale GaN film as a spacer; the entire structure is coated on the back side with a gold film. The resonator consists of a split strip and a patch, a bias wire across the HEMT to serve as a gate, and the source and drain wires located on either side of the resonator. The 2DEG is formed of an AlGaN/GaN heterostructure nested in the gap of the split strip; this forms a 2DEG layer based on spontaneous and piezoelectric polarization effects within the heterostructure. The gate and source-drain electrodes connect between the heterostructure and gold pattern by a Schottky contact and Ohmic contacts, respectively. By applying bias voltages to control carrier concentration within 2DEG, large amplitude and phase modulation of an incident free-space THz wave can be realized. Moreover, the geometric asymmetry of the meta-elements due to the rectangular patch on one side of the unit cell can induce a significant asymmetry in the currents on the source and drain electrodes, which varies depending on the carrier density of the 2DEG channel. Our design exploits this asymmetry to optimize the amplitude and phase modulation of the reflected wave, as shown below. To achieve spatial control of wave fronts, we group all the meta-elements along a line (parallel to the *y*-axis) into single-column subarrays sharing a common bias line (Fig. 1c) which can be controlled via a coding sequence from an external field-programmable gate array (FPGA). Meanwhile, both the source and drain electrodes of all of the meta-elements are connected to a common ground line which runs across the center of the metasurface (parallel to the *x-*axis). This bias-wire layout simplifies the control network, whereby all unit cells in each column are controlled with identical external voltage, thus remaining in phase. With the *x*-axis of symmetry defined by the central ground line, we create two mirrored subarrays on the +*y* and -*y* sides, to enable quasi-2D phase control. Compared with one-dimensional (1D) column-wise feeding^16,27^, this dual-region mirrored subarray configuration provides more flexibility without redundant circuit coupling.

**Fig. 2:**
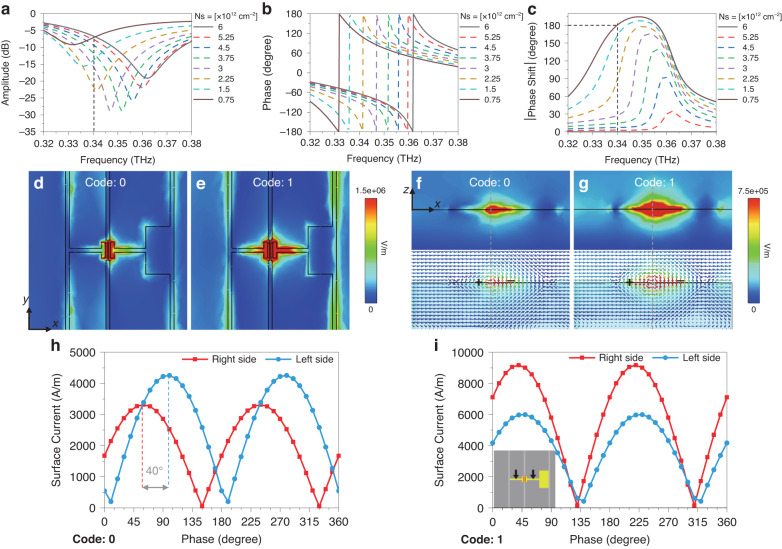
Simulations of the meta-element based on 2DEG-regulated asymmetric resonance. **a**–**c** The amplitudes, phase, and phase shift with the variation of carrier concentration, respectively. **d**, **e** Top and **f**, **g** section views of electric-field distributions at 0.34 THz for the "0" and "1" coding states, respectively. **h**, **i** A period of current intensity at the central points of the right strip and left strip, at 0.34 THz for the "0" and "1" coding states, showing a phase delay in the "0" case because of the asymmetric resonance

As a first step towards understanding the quasi-optical behavior of this structure, we simulate the reflection amplitude and phase responses of a meta-element with a period *d* by Floquet ports and unit-cell boundary in CST Microwave Studio. The dispersion function for the 2DEG layer is expressed by the classic Drude model (detailed in Supplementary Note 1) to describe variations of carrier concentration *N*_*s*_. After extensive simulations to explore the parameter space, we extract the optimized geometric dimensions of the meta-element as follows: *d* = 200 μm, *h* = 200 μm, *l* = 40 μm, *a* × *b* = 30 × 60 μm^2^, and *w* = 5 μm. Figure 2 shows the simulated results using these values, assuming an incident *x*-polarized plane wave with a 15° oblique angle of incidence. Results are shown for the amplitude response (Fig. 2a) and phase response (Fig. 2b) as a function of the carrier density *N*_*s*_ of the 2DEG. These results manifest a dramatic resonant response in both amplitude and phase, with a broadband smooth phase shift exceeding 150° in the range of 0.334–0.36 THz. This resonance shifts significantly to lower frequencies as *N*_*s*_ decreases. In Fig. 2c, we plot the phase difference ϕ(*N*_*s*_^on^) – ϕ(*N*_*s*_^off^), where ϕ(*N*_*s*_^on^) is the phase of the reflected wave at carrier density *N*_*s*_^on^, and *N*_*s*_^off^ is a reference value of 6 × 10^12 ^cm^−2^ (therefore, the curve in this figure corresponding to *N*_*s*_^off^ = 6 × 10^12 ^cm^−2^ is simply zero at all frequencies). This plot, along with the amplitude response in Fig. 2a, reveals a remarkable result: at a particular frequency of 0.34 THz and for a particular value of *N*_*s*_^on^ = 0.75 × 10^12 ^cm^−2^, the phase difference reaches 180° while the amplitude responses are equal (see dashed vertical lines in Fig. 2a, c). Besides, this result has a good tolerance under different incident angles within 30°, which is detailed in Supplementary Note 2. We may therefore consider these two values of the 2DEG doping to correspond to the ideal "on" and "off" states in a 1-bit coding operation, with a uniform reflectance and 180° phase shift at the chosen operating frequency. The values of *N*_*s*_ = 0.75 × 10^12 ^cm^−2^ and 6.0 × 10^12 ^cm^−2^ thus correspond to digital code values "1" and "0", respectively.

In order to clarify the significance of the geometrical asymmetry, we show in Fig. 2d–i the distributions of the electric field and surface current with an incident field at 0.34 THz, under the two bias conditions corresponding to the digital "1" and "0". These simulations illustrate the strong asymmetry of the resonant electric field distribution when the 2DEG contains more carriers (digital "0"), in contrast to the much more symmetric situation which results when the 2DEG is depleted under a bias voltage (digital "1"). The 2DEG acts as a connection between the resonator gap. Strong connectivity in the state "0" enables free electrons in resonant current to flow through the gap. However, the patch on the right side makes its impedance lower than the left side, which leads the electrons to accumulate at the low-impedance side when flowing from the high-impedance side. Therefore, an asymmetric electric field distribution (in Fig. 2d, f) can be created to cause a resonant phase delay between both sides (Fig. 2h). Conversely, when the 2DEG is depleted in the state "1", free electrons will be blocked at the two gap sides simultaneously, exhibiting a nearly symmetric resonant field (Fig. 2e, g) and in-phase resonant current (Fig. 2i). This modulation for the current flow asymmetry (and the associated electromagnetic asymmetry) is not only reflected in these near-field simulations; they also imprint signatures on the amplitude and phase of a free-space wave reflected from the device, which therefore shows a strong dependence on the 2DEG bias condition as discussed above and shown in Fig. 2a–c.

### Multifunctional phase-coding strategies

Next, with the understanding of an individual meta-element discussed above, we now turn to considerations of the entire multi-column metasurface. By controlling the 1-bit digital state of each of the sub-groups of meta-elements (see Fig. 1c), we can engineer the near-field phase distribution (and therefore the far-field wave front) of the reflected THz wave (see Supplementary Eq. (S3) for details). Here, we consider several possible coding matrices, and perform numerical predictions of the output beam patterns that would result. We assume a metasurface consisting of 64 × 64 elements with *d* = 200 μm, operating at 0.34 THz (corresponding to the experimentally realized device discussed below). To accelerate the computation for this large array, we regard each element as an ideal isotropic radiator, and we neglect the effects of near-field coupling of nearby elements.

**Fig. 3:**
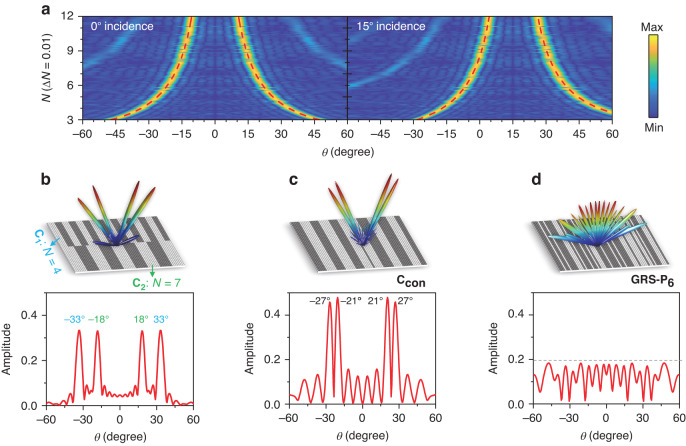
Numerical calculations for multifunctional coding-beam manipulations at 0.34 THz. **a** Nearly continuous beam scanning based on fractional coding. **b** Multi-beam beam steering with the dual-region coding matrixes **C**_**1**_, **C**_**2**_, and **c** convolutional coding matrix **C**_**con**_. **d** Diffuse scattering based on the P_6_-type GRS coding

According to the generalized Snell’s law, the elevation angle *θ* of the main lobe on a 1D 1-bit metasurface can be predicted as

1 $$\theta =\arcsin (\frac{\pm {\rm{\pi }}+k\cdot Nd\,\sin {\theta }_{i}}{k\cdot Nd})$$

where ±π denotes the phase difference between "0" and "1" states for 1-bit coding; *d* is a single element period and *N* is the number of adjacent in-phase elements. This result was originally envisioned for integer values of *N*, which would limit the angle of the main lobe to a few discrete values. To overcome this limitation and realize quasi-continuous beam scanning, we have recently proposed a fractional phase-coding strategy^34^. Fractional coding gives an encoding rule for *N* taking any non-zero positive value, not just integers (see Supplementary Note 3). This enables a higher degree of angular precision, and can still be denoted by the corresponding values of *N*. As a demonstration of the capability of quasi-continuous beam steering, we compute the 1D phase-gradient distributions for the metasurface under consideration here, and plot the corresponding beam patterns in Fig. 3a for different values of *N*. As the 1-bit coding varies from *N* = 3 to *N* = 12 (with the interval Δ*N* = 0.01), in the view field from −60° to 60°, nearly continuous dual-beam scanning is obtained between −47.4°–−10.7° and 10.7°–47.4° under a normal incident input beam. This range shifts to −28.5°–4.2° and 26.4°–60° under a 15° oblique incidence. The directivity of the main lobe is consistent with the prediction (the red dashed curves) from Eq. (1), reflecting a non-linear relation between *N* and *θ*. As the beam angle increases, *N* decreases slowly with a smaller Δ*N* for the same beam-scanning interval Δ*θ* and vice versa. On the other hand, more continuous beam scanning can be achieved by taking a smaller Δ*N* to obtain a subtle phase-gradient variation, and the dynamic range of beam steering can be enlarged by expanding the range of *N*.

As a next step, we implement multi-beam steering with the proposed metasurface through dual-region coding and convolutional coding^35^. The former regulates multi-beam patterns by subdividing the metasurface into more than one independent coding region. The latter synthesizes a beam pattern with another direction by the Fourier operation of two coding matrixes. Considering normal plane-wave incidence, Fig. 3b–d illustrate several possible beam patterns. Here, the coding matrices are plotted as gray-white bicolor arrays to represent the 1-bit phase distributions. For dual-region beam steering (Fig. 3b), we apply two 32 × 64 coding matrices on the upper and lower halves of the metasurface, to achieve centrosymmetric dual-beam reflections at ±33° and ±18° via the coding matrixes **C**_**1**_ with *N* = 4 and **C**_**2**_ with *N* = 7, respectively. For convolutional coding, Fig. 3c shows a quad-beam pattern at −24° ± 3° and 24° ± 3° whose convolutional coding matrix **C**_**con**_ is produced by the Fourier operation (see Supplementary Note 3. (2)) between the **C**_**a**_ with *N* = 32 and **C**_**b**_ with *N* = 5.5. The angles are consistent with the equation *θ* = sin^−1^(sin*θ*_1_+sin*θ*_2_), where *θ*_1_ = ± 3° and *θ*_2_ = ± 24° respectively generated by **C**_**a**_ and **C**_**b**_ (the beam patterns of **C**_**a**_ and **C**_**b**_ are shown in Supplementary Fig. S4a). The reflection amplitude of the main lobe depends on the size of coding subarrays for dual-region coding. We note that convolutional coding more efficiently utilizes the coding resources to concentrate beam energy towards the main lobe, leading to a greater main-lobe amplitude than the dual-region coding case. However, the sidelobe levels of convolutional coding are slightly higher than dual-region coding due to greater coherence between the array elements.

Finally, we investigate the possibility of creating a diffusely scattered wave front, using an aperiodic coding sequence. Such diffuse wave fronts can be valuable in numerous imaging applications, but the process of generating these cannot simply rely on a random sequence of coding values. Rather, to ensure that the diffuse wave exhibits no local maximum that is significantly above the mean, a typical approach often relies on brute-force numerical optimization to create a particular sequence of control signals^36–39^, a computationally demanding procedure. Instead, we rely on an alternative approach recently described in ref. ^40^, which relies on Golay-Rudin-Shapiro (GRS) polynomials to impose a flatness criterion. This GRS coding approach is a deterministic and computationally inexpensive algorithm which is compatible with a continuous spectral response^40^. In the case of maximum available length on a 64 × 64 metasurface, Fig. 3d shows the 1-D result obtained from GRS coding; this result effectively diffuses the reflected beams and suppresses the maximum reflectance to <20% (details of the GRS coding computation are found in Supplementary Note 3. (3)).

### Experimental demonstration

**Fig. 4:**
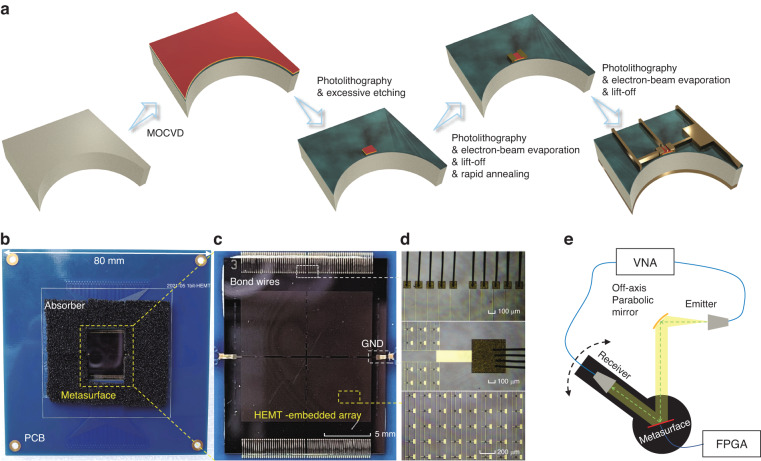
Preparation for the experimental demonstration. **a** Process flow for the fabrication of the coding metasurface. **b**–**d** The close-up and microscope photos of the real metasurface. **e** The test platform for beam measurements based on a VNA system

We fabricated and characterized a proof-of-concept prototype of the proposed metasurface, as shown in Fig. 4. The metasurface consists of 64 × 64 meta-elements with a total area of 13 × 13 mm^2^, in which every 1 × 32 meta-elements compose one single-column subarray. The device is mounted on a PCB, and then the bias- and ground-electrode pads are connected to the external circuits by gold-wire bonds. To avoid onboard interference, a piece of absorbing material is paved around the metasurface on the PCB. Figure 4b–d show close-up and microscope photos of the fabricated device. We characterize the device by illuminating it with a THz wave at a 15° oblique angle of incidence, and measuring the reflected wave in the angular range of 15°–60° (limited by the size of the test platform used to hold the receiver). The test platform consists of a THz vector network analyzer (VNA), a parabolic mirror for the plane-wave excitation, an FPGA control module, T/R modules, and a rotary stage, as illustrated in Fig. 4e. Following the analysis discussed above, we implement several different functions including beam scanning, multi-beam generation, and diffuse scattering.

**Fig. 5:**
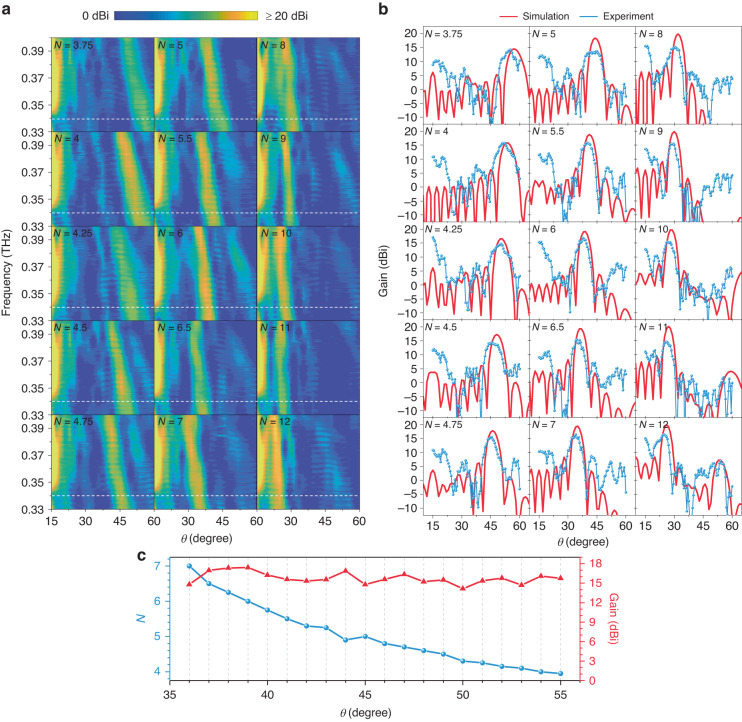
Measured results for beam scanning with N changed from 3.75 to 12. **a** The wide-band beam scanning from 60° to 20° at 0.33–0.4 THz. **b** Comparison between the 1-D results extracted from **a** (blue curves) and simulations (red curves) at 0.34 THz. **c** The measured high-precision beam scanning with a 1° step length

To account for the path loss, the beam measurements are normalized by replacing the metasurface with a flat metallic sheet of the same size. With the coding sequences (listed in Supplementary Table S1) ranging from *N* = 3.75 to *N* = 12, the measured results for beam scanning are shown in Fig. 5. The results show wide-band quasi-continuous beam scanning with a 70 GHz bandwidth. As shown in Fig. 5a, the beam angle shifts with the coding sequences and also varies with frequencies in the range of 0.33–0.4 THz. Moreover, the beam scanning covers the angle range from 20° to 60°. Around the central working frequency of 0.34 THz, the specular reflections at 15° are the weakest (marked by white dashed lines in Fig. 5a) attributed to the amplitude-phase states that are near to the ideal 1-bit condition. As the amplitude-phase states deviate from the ideal 1-bit situation at other frequencies, the specular beams become conspicuous accordingly while the main lobes can still be maintained. Figure 5b extracts the single-frequency results (blue curves) at 0.34 THz from Fig. 5a and compares them with the simulations (red curves). The beam directivity agrees well with the simulations with no angular error >2°, and a mean angular error of 1.3°. Using a more elaborate fractional coding with a smaller Δ*N*, we can achieve a scanning accuracy of 1° in the range from 35° to 55° (Fig. 5c).

Although there is good agreement between measurements and simulations, our results do exhibit some discrepancies. Due to the edge effects^41^, we expect that the beamwidth should decrease as the reflection angle decreases, and correspondingly the directional gain should increase. According to our simulations, the half-power beamwidth (HPBW) decreases from 8° to 5° with the beam scanning from 57° to 26°, and the gain correspondingly increases from 15 dBi to 20 dBi. However, the measured gain does not increase with a smaller beam angle, which leads to a 5 dBi maximum error with the simulations. Moreover, we observe that the specular reflections in the measured results are predicted to be much smaller in the simulations. We attribute these non-ideal behaviors to fabrication errors, such as non-uniformities of the epitaxial layer and the connection of electrodes, as well as to other factors including imperfect optical-path collimation, leakage-wave interference, and VNA sensitivity.

**Fig. 6:**
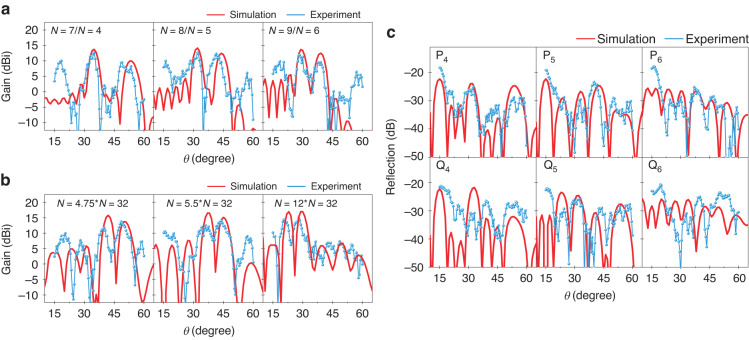
Measured results for dual-beam manipulations and diffuse scattering at 0.34 THz. **a** The beam patterns with the dual-region coding combinations: *N* = 7/*N* = 4, *N* = 8/*N* = 5, and *N* = 9/*N* = 6, respectively. **b** The beam patterns with the convolutional coding combinations: *N* = 4.75*N = 32*, N* = 5.5*N = 32*, and N* = 12*N = 32, respectively. **c** The scattering-field patterns with the GRS coding sequences: P_4_, P_5_, P_6_, Q_4_, Q_5_, and Q_6_, respectively

Figure 6a, b show the measurement results of dual-region and convolutional coding at 0.34 THz. A dual-region coding sequence synthesized by *N* = *n*_1_ and *N* = *n*_2_ is labeled as "*N* = *n*_1_/*N* = *n*_2_" in Fig. 6a. The three coding combinations (listed in Supplementary Table S2): *N* = 7/*N* = 4, *N* = 8/*N* = 5, and *N* = 9/*N* = 6 control respectively the dual-beam directivities at 35°&51°, 31°&42°, and 28.5°&38.5° in the experiments. It should be noted that the peak of the second beam at 51° (controlled by *N* = 4) appears to be a clipping defect. In contrast, the first beam at 35° (controlled by *N* = 7) has not been influenced by the clipping effect. The beams do not interfere with each other owing to the good isolation between the two coding regions.

In Fig. 6b, a convolutional coding sequence after the Fourier operation by *N* = *n*_1_ and *N* = *n*_2_ is labeled as "*N* = *n*_1_*N = *n*_2_". The dual-beam directivities at 40° and 48.5°, 36° and 44°, and 21° and 28° are regulated and measured by the coding combinations (listed in Supplementary Table S3): *N* = 4.75*N = 32, *N* = 5.5*N = 32, and *N* = 12*N = 32, respectively. As mentioned in the above discussion, convolutional coding concentrates main-lobe energy but raises sidelobe levels; this behavior can be observed in both the simulations and measurements. Convolutional coding achieves a 14.8 dBi maximum gain (>12.6 dBi gain by dual-region coding), and shows higher sidelobe levels. Compared with the simulations, the directivity errors are both <2° for the two coding methods, and the gain errors are <2.5 dBi for dual-region coding and <4 dBi for convolutional coding. Next, we test the ability for diffuse scattering by P_*v*_- and Q_*v*_-type GRS coding sequences (listed in Supplementary Table S4) with the lengths *v* = 4, 5, and 6, as shown in Fig. 6c. Since GRS coding as a kind of quasi-random sequence has harsh fault tolerance, the specular lobes at 15° caused by the above error factors weaken the ability of diffuse scattering. However, the maximum reflectivity is still below ~−20 dB and the directivities are almost in accord with the simulations other than the specular reflections.

### Point-to-point tracking transmission

**Fig. 7:**
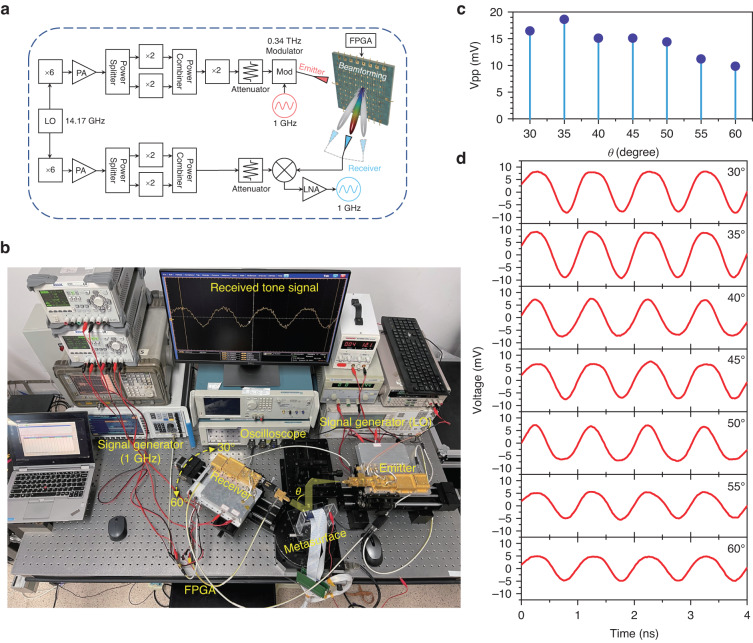
THz metasurface-aided point-to-point signal transmission based on real-time beam tracking. **a** The diagram and **b** real photo of the transmission link based on a direct-modulation THz communication system at 0.34 THz. **c** V_pp_ of the real-time modulated single-tone signal and **d** the waveforms in different directions

As a final demonstration of the versatility of this reconfigurable metasurface, we demonstrate a tracking transmission system (see Supplementary Video). This proof-of-principle experiment relies on a point-to-point transmission of a 1 GHz single-tone signal at 0.34 THz. As shown in Fig. 7a, the basic THz communication link employs a direct modulation technique. The emitter is composed of a frequency multiplier module and a modulator module. First, W-band waves are generated via a 6× frequency multiplier driven by a 14.17 GHz local oscillator (LO). These W-band waves are input into two 2× multipliers after power amplifying and power splitting to produce 0.17 THz waves. Next, we obtain carrier waves at 0.34 THz via the last 2× multiplier and then input them into a THz modulator after power matching by an attenuator. Finally, a signal generator inputs a 1 GHz single-tone signal into the modulator to transmit modulated waves. For the receiver, a 0.34 THz second harmonic mixer driven by 0.17 THz waves implements zero-intermediate-frequency demodulation to recover the 1 GHz single-tone signal. The receiver on a rotating platform can be positioned in azimuth from 30° to 60° to mimic targets in different directions, as shown in Fig. 7b. For a demonstration of beam tracking, we turn this rotating platform with a 5° increment. We observe that the link can be closed only when a beam is pointed to the receiver. By using the known receiver angle to determine the appropriate value of *N* (using Eq. (1)), we can apply the corresponding coding sequence to the metasurface, to produce a reflected beam directed towards the receiver. The smoothed demodulated results in different directions are shown in Fig. 7c, d. As the receiver turns from 30° to 60°, the demodulated signal retains a clear 1 GHz sine wave, although the peak-to-peak voltage V_pp_ shows a downtrend owing to the inevitable edge coherence effect mentioned above. Slight waveform distortion results from high-order nonlinear harmonics of diodes within the modulator. With a suitable location sensing capability to automate this process^42^, this beam tracking functionality could be implemented in real time, as the switching speed of the metasurface can reach to 100 MHz (see Supplementary Note 6).

## Discussion

The idea of using intelligent reconfigurable surfaces to enhance the capabilities of imaging, communications, and sensing systems in the millimeter-wave and THz regimes has been a subject of considerable interest^43–47^, especially in the last few years. Although the value of such devices is clear, the implementation has remained uncertain, given the many stringent demands on their performance specifications. Here, we have proposed and experimentally verified a real-time controlled THz programmable metasurface based on a carefully designed asymmetric meta-element with a 2DEG switch, which can simultaneously satisfy many of these operation criteria for fast and agile wavefront manipulation. By changing carrier concentration in the 2DEG with an applied bias, the resonant delay imposed on an incident wave can be switched by 180° with a uniform amplitude, thus permitting the definition of digital on and off states (i.e., a 1-bit coding). A numerical beam-pattern analysis, together with experimental studies, demonstrates the use of this structure for multifunctional beam steering and wave front engineering, enabling many applications in which a smart reconfigurable surface is required. For example, in a view field from 20° to 60°, we realize a wide-band beam scanning in 0.33–0.4 THz with a 1° scanning accuracy at 0.34 THz. For creating a diffusely scattered wave, we employ GRS coding to obtain a measured maximum reflection of ~−20 dB. We also implement real-time beam tracking to verify the metasurface-assisted point-to-point signal transmission in different directions. Compared with THz programmable metasurfaces recently proposed (listed in Table 1), this work provides a balanced configuration without additional phase shifters for good response speed and beam-scanning precision. In the future, larger-scale and higher-performance THz metasurfaces can be extended based on the results presented here, and may be applied in THz high-speed wireless communications, super-resolution imaging systems, and other advanced applications.

## Materials and methods

### Calculation of coding sequences

The phase distributions on the metasurface determine the coding sequences, which are designed by the numerical approaches detailed in Supplementary Note 3. All coding sequences used in the experiments are listed in Supplementary Tables S1 to S4.

### Metasurface fabrication

Figure 4a illustrates the fabrication process. Firstly, an AlGaN/GaN epitaxial layer is grown on a SiC substrate by metalorganic chemical vapor deposition (MOCVD). After a standard cleaning process and photolithography processes, the active region of 2DEG is delimited with the outside AlGaN layer being etched by inductively coupled plasma. Next, the source-drain electrodes are formed by photoetching, electron-beam evaporation, lift-off processes, and rapid annealing at 900 °C under an N_2_ environment, with Ti/Al/Ni/Au deposition on both ends of the active region as ohmic contacts. Similarly, for meta-atom patterning, Ni/Au layer is fabricated on the GaN film by similar photo etching, electron-beam evaporation, and lift-off processes.

### Beam measurements

THz waves from the emitter impinge on the metasurface with a 15° oblique angle after being collimated by the off-axis parabolic mirror. The metasurface is located at the axis of a rotating platform and reflects THz waves after digitalized processing by a series of coding sequences from the FPGA, where the binary codes "0" and "1" correspond to the states of −8 V bias and 0 V bias (which are determined by the 1-bit condition at the central operating frequency of 0.34 THz, detailed in Supplementary Note 5. (2)). The receiver on the rotating platform turns in the azimuth from 15° to 60° with a 0.5° increment to measure reflection beam patterns. Because our emitter produces limited output power, the receiver is located only 15 cm from the metasurface.

### Supplementary information

Supplementary information for Real-time programmable metasurface for terahertz multifunctional wave front engineering

Point-to-point beam tracking transmission

## References

[1] Kürner T, Priebe S (2014). Towards THz communications—status in research, standardization and regulation. J. Infrared Millim. Terahertz Waves.

[2] Koenig S (2013). Wireless sub-THz communication system with high data rate. Nat. Photonics.

[3] Federici JF (2005). THz imaging and sensing for security applications—explosives, weapons and drugs. Semicond. Sci. Technol..

[4] Cooper KB (2011). THz imaging radar for standoff personnel screening. IEEE Trans. Terahertz Sci. Technol..

[5] Fischer BM, Helm H, Jepsen PU (2007). Chemical recognition with broadband THz spectroscopy. Proc. IEEE.

[6] Wang Q, Xie LJ, Ying YB (2022). Overview of imaging methods based on terahertz time-domain spectroscopy. Appl. Spectrosc. Rev..

[7] Yang X (2016). Biomedical applications of terahertz spectroscopy and imaging. Trends Biotechnol..

[8] Huang, C. W. et al. Hybrid beamforming for RIS-empowered multi-hop terahertz communications: a DRL-based method. In *2020 IEEE Globecom Workshops* (GC Wkshps, IEEE, 2020).

[9] Han C, Yan LF, Yuan JH (2021). Hybrid beamforming for terahertz wireless communications: challenges, architectures, and open problems. IEEE Wirel. Commun..

[10] Kannegulla A (2014). Coded-aperture imaging using photo-induced reconfigurable aperture arrays for mapping terahertz beams. IEEE Trans. Terahertz Sci. Technol..

[11] Luo CG (2019). High-resolution terahertz coded-aperture imaging for near-field three-dimensional target. Appl. Opt..

[12] Sato K, Monnai Y (2020). Terahertz beam steering based on trajectory deflection in dielectric-free Luneburg lens. IEEE Trans. Terahertz Sci. Technol..

[13] Yang Y, Gurbuz OD, Rebeiz GM (2016). An eight-element 370-410-GHz phased-array transmitter in 45-nm CMOS SOI with peak EIRP of 8–8.5 dBm. IEEE Trans. Microw. Theory Tech..

[14] Wu JB (2020). Liquid crystal programmable metasurface for terahertz beam steering. Appl. Phys. Lett..

[15] Liu CX (2021). Programmable manipulations of terahertz beams by transmissive digital coding metasurfaces based on liquid crystals. Adv. Opt. Mater..

[16] Karl N (2014). An electrically driven terahertz metamaterial diffractive modulator with more than 20 dB of dynamic range. Appl. Phys. Lett..

[17] Venkatesh S (2020). A high-speed programmable and scalable terahertz holographic metasurface based on tiled CMOS chips. Nat. Electron..

[18] Monroe, N. M. et al. Electronic THz pencil beam forming and 2D steering for high angular-resolution operation: a 98 × 98-unit 265GHz CMOS reflectarray with in-unit digital beam shaping and squint correction. In *2022 IEEE International Solid- State Circuits Conference (ISSCC)* 1–3 (IEEE, CA, 2022).

[19] Lou J (2020). Silicon-based terahertz meta-devices for electrical modulation of Fano resonance and transmission amplitude. Adv. Opt. Mater..

[20] Lou J (2021). Optically controlled ultrafast terahertz metadevices with ultralow pump threshold. Small.

[21] Lou J (2022). Calibration-free, high-precision, and robust terahertz ultrafast metasurfaces for monitoring gastric cancers. Proc. Natl Acad. Sci. USA.

[22] Lin CH (2021). Automatic inverse design of high-performance beam-steering metasurfaces via genetic-type tree optimization. Nano Lett..

[23] Lin QW (2022). Coding metasurfaces with reconfiguration capabilities based on optical activation of phase‐change materials for terahertz beam manipulations. Adv. Opt. Mater..

[24] Zhao YC (2018). Dynamic photoinduced controlling of the large phase shift of terahertz waves via vanadium dioxide coupling nanostructures. ACS Photonics.

[25] Kim Y (2019). Phase modulation with electrically tunable vanadium dioxide phase-change metasurfaces. Nano Lett..

[26] Pitchappa P (2019). Chalcogenide phase change material for active terahertz photonics. Adv. Mater..

[27] Chen BW (2022). Electrically addressable integrated intelligent terahertz metasurface. Sci. Adv..

[28] Zhao YC (2019). High-speed efficient terahertz modulation based on tunable collective-individual state conversion within an active 3 nm two-dimensional electron gas metasurface. Nano Lett..

[29] Zhang YX (2018). Large phase modulation of THz wave via an enhanced resonant active HEMT metasurface. Nanophotonics.

[30] Zeng HX (2021). High-precision digital terahertz phase manipulation within a multichannel field perturbation coding chip. Nat. Photonics.

[31] Hunt J (2013). Metamaterial apertures for computational imaging. Science.

[32] Li YB (2016). Transmission-type 2-bit programmable metasurface for single-sensor and single-frequency microwave imaging. Sci. Rep..

[33] Gan FJ (2022). Robust compressive terahertz coded aperture imaging using deep priors. IEEE Geosci. Remote Sens. Lett..

[34] Wang LY (2020). A fractional phase-coding strategy for terahertz beam patterning on digital metasurfaces. Opt. Express.

[35] Liu S (2016). Convolution operations on coding metasurface to reach flexible and continuous controls of terahertz beams. Adv. Sci..

[36] Dong DS (2015). Terahertz broadband low-reflection metasurface by controlling phase distributions. Adv. Opt. Mater..

[37] Gao LH (2015). Broadband diffusion of terahertz waves by multi-bit coding metasurfaces. Light Sci. Appl..

[38] Zhao Y (2016). Broadband diffusion metasurface based on a single anisotropic element and optimized by the simulated annealing algorithm. Sci. Rep..

[39] Li SJ (2016). Ultra-broadband reflective metamaterial with RCS reduction based on polarization convertor, information entropy theory and genetic optimization algorithm. Sci. Rep..

[40] Moccia M (2017). Coding metasurfaces for diffuse scattering: scaling laws, bounds, and suboptimal design. Adv. Opt. Mater..

[41] Nayeri P, Yang F, Elsherbeni AZ (2013). Bifocal design and aperture phase optimizations of reflectarray antennas for wide-angle beam scanning performance. IEEE Trans. Antennas Propag..

[42] Ghasempour Y (2020). Single-shot link discovery for terahertz wireless networks. Nat. Commun..

[43] Menzel W, Pilz D, Al-Tikriti M (2002). Millimeter-wave folded reflector antennas with high gain, low loss, and low profile. IEEE Antennas Propag. Mag..

[44] Li LL (2022). Intelligent metasurfaces: control, communication and computing. eLight.

[45] Ma Q (2022). Directly wireless communication of human minds via non-invasive brain-computer-metasurface platform. eLight.

[46] Dai LL (2020). Reconfigurable intelligent surface-based wireless communications: antenna design, prototyping, and experimental results. IEEE Access.

[47] Yang FY, Pitchappa P, Wang N (2022). Terahertz reconfigurable intelligent surfaces (RISs) for 6G communication links. Micromachines.

[48] Lynch, J. J. et al. Coded aperture subreflector array for high resolution radar imaging. In *Proc. SPIE 10994, Passive and Active Millimeter-Wave Imaging XXII* 1099405 (SPIE, 2019).

