# Supplementary material for: Real-time programmable metasurface for terahertz multifunctional wave front engineering
Source: Light Sci Appl. 2023 Aug 7;12:191. doi: 10.1038/s41377-023-01228-w (PMC10406829; doi:10.1038/s41377-023-01228-w)
Supplement: Supplementary file 1 — Supplementary information for Real-time programmable metasurface for terahertz multifunctional wave front engineering [file 41377_2023_1228_MOESM1_ESM.pdf]

Supplementary Information for

Real-time programmable metasurface for terahertz multifunctional wave front engineering

Feng Lan[1,4,9], Luyang Wang[1,9], Hongxin Zeng[1,], Shixiong Liang[3], Tianyang Song[1], Wenxin Liu[5,6], Pinaki Mazumder[7], Ziqiang Yang[1,4], Yaxin Zhang[1,2,4,*] and Daniel M. Mittleman[8,*]*

[1] Sichuan THz Communication Technology Engineering Research Center, School of Electronic Science and Engineering, University of Electronic Science and Technology of China, Chengdu 611731, China.
[2] Zhangjiang Laboratory, Shanghai 201204, China.
[3] National Key Laboratory of Application Specific Integrated Circuit, Hebei Semiconductor Research Institute, Shijiazhuang 050051, China.
[4] Yangtze Delta Region Institute (Huzhou), University of Electronic Science and Technology of China, Huzhou 313000, China.
[5] Aerospace Information Research Institute, Chinese Academy of Sciences, Beijing 100094, China.
[6] University of Chinese Academy of Sciences, School of Electronic, Electrical and Communication Engineering, Beijing 101408, China.
[7] Department of Electrical Engineering and Computer Science, University of Michigan, Ann Arbor, MI 48109, USA.
[8] School of Engineering, Brown University, Providence, RI 02912, USA.
[9] These authors contributed equally to this work: Feng Lan and Luyang Wang.

[*] Corresponding authors:
Yaxin Zhang(zhangyaxin@uestc.edu.cn);
Hongxin Zeng(zenghx@uestc.edu.cn);
Daniel M. Mittleman (daniel_mittleman@brown.edu)

Note 1. Dispersion model of the 2DEG layer

We adopt the Drude model, also known as the cold-plasma model, to describe the dispersion characteristics of the 2DEG layer with varying carrier concentrations[1,2]. The collision frequency v_c is introduced to describe damping generated by elastic collisions of the moving electrons with the stationary particles. Regarding the specific plasma frequency ω_p, the complex relative permittivity can be expressed as

$$\varepsilon(\omega) = \varepsilon_\infty - \frac{\omega_p{}^2}{\omega(\omega - \mathrm{i}v_c)} \tag{S1}$$

The collision frequency v_c=3.44×10^{12} Hz is calculated with the relation v_c=$e/m^*\mu$, in which e and m^* are the electron charge and effective mass in the 2DEG layer, respectively. According to the epitaxial material datasheet, the electron mobility μ is 2321 cm^2·(V·s)$^{-1}$. The plasma frequency ω_p and the two-dimensional carrier concentration N_s meet the relation as

$$\omega_p = \sqrt{\frac{N_s e^2}{\varepsilon_0 m^*}} \tag{S2}$$

Based on the above Drude model, we can simulate the 2DEG depletion process controlled by external bias voltages by decreasing the carrier concentration.

Note 2. Tolerance simulation to different incident angles

According to the simulated amplitude-phase modulation of the proposed coding element, the metasurface can realize beam steering with negligible deteriorations under a normal and oblique incidence within 30°. Corresponding amplitude-phase responses and deflected angles of beam scanning are analyzed in what follows.

Fig. S1 simulates the amplitudes and phase responses with the incident angular variation from 0° to 50°. Acceptable 1-bit-coding condition of the near uniform amplitude and phase shift around 180° can be maintained with the incident angles of 0°–30°. Further increasing the incident angle up to 50°, the phase shift deviates over 20° away from 180° since the increasing horizontal components undermine the resonance intensity that is needed by a phase shift of 180°.

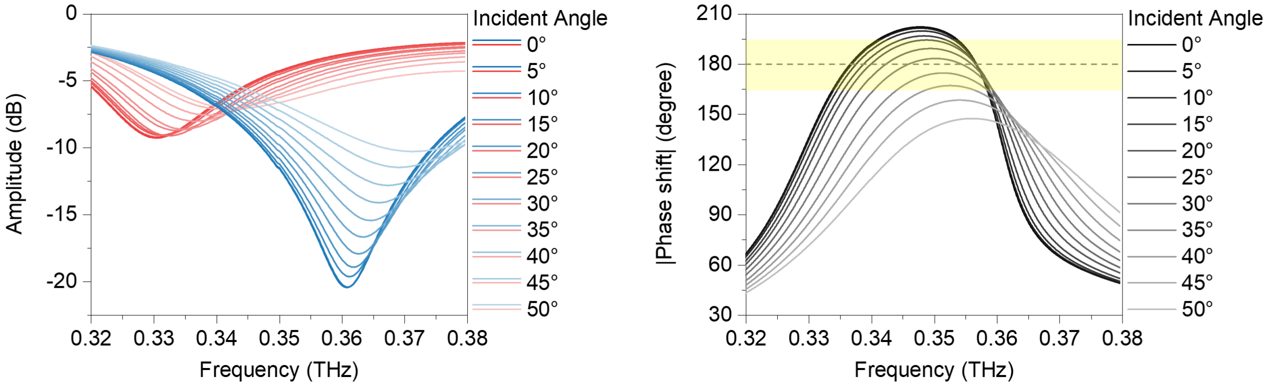

Fig. S1 The responses of amplitude and phase with different incidence angles.

Note 3. Numerical calculations for coding strategies and beam patterns

According to phased reflectarray theory, under the illumination from a direction of (θ_i, φ_i), a normalized beam pattern for an X×Y reflectarray can be expressed as

$$F(\theta,\varphi)=\frac{f_{\mathrm{e}}(\theta,\varphi)}{X\cdot Y}\times\sum_{x=1}^{X}\sum_{y=1}^{Y}e^{-\mathrm{j}(\varphi(x,y)+kd(x-1)(\sin\theta\cos\varphi-\sin\theta_{\mathrm{i}}\cos\varphi_{\mathrm{i}})+kd(y-1)(\sin\theta\sin\varphi-\sin\theta_{\mathrm{i}}\sin\varphi_{\mathrm{i}}))} \qquad \text{(S3)}$$

Where $f_e(\theta,\varphi)$ is an element pattern function; k is a vacuum wavenumber; $\varphi(x, y)$ is an element phase response; and d is a single element period, respectively. To convert the rigid phase-gradient beam manipulations into flexible digital beam synthesis, we encode each element with binary numbers for different phase responses to establish a mapping relation between phase distributions and coding matrixes.

(1) Beam Scanning based on fractional coding

In Eq. (1), the discrete phase gradient forms by a periodic coding sequence for N=n combines n "0" s and n "1" s. For instance, N=1, 2, and 3 can be encoded as {...0 1 0 1...}, {... 0 0 1 1...}, and {...0 0 0 1 1 1...}. Since a value of N can only take a positive integer for the entitative unit cell, the rough discrete precision of phase distributions limits the directivity accuracy of beam steering. Fractional coding can achieve quasi-continuous beam scanning and accurately directed beams by breaking the limitation of integer N.

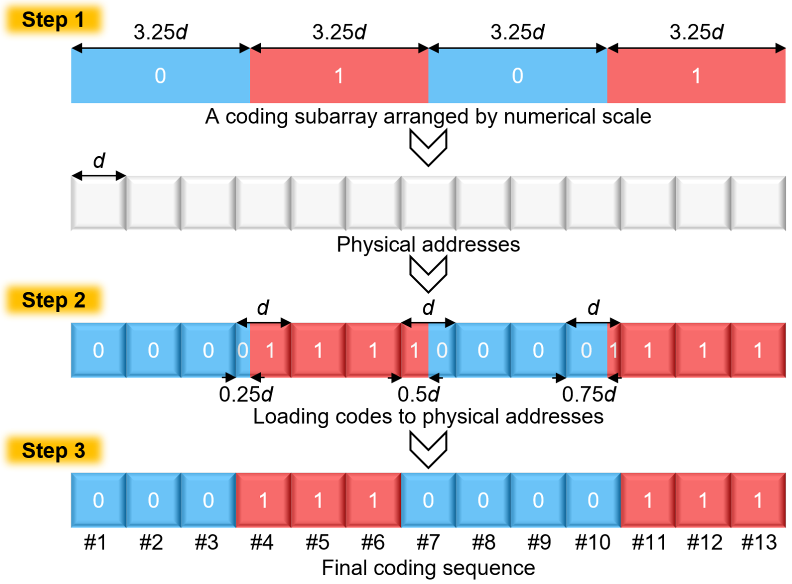

Fig. S2 Discrete process of the fractional coding method.

Tacking the 1-bit coding case with N=3.25 as an example, Fig. S2 shows the general scheme of the fractional coding method. In step 1, we suppose the dimension of an in-phase period is 3.25d, which forms a subarray including the 1-bit coding states “0” and “1” (differentiated by blue and red rectangles). In step 2, the logical coding sequences will be applied to the physical unit cell one by one. In step 3, two coding states in one unit are recoded by choosing the major filling factor as the final state of this unit. More specifically, *Code*(4) is recoded as “1” which is the major filling factor accounting for 75%, as shown in Fig. S2. For the half to half situation, *Code*(7) can be any state due to two codes both accounting for 50%. Similarly, *Code*(10) is confirmed as “0”. The final periodic coding sequence for N=3.25 is {...0 0 0 1 1 1 0 0 0 0 1 1 1...}. According to this method, we define the filling factor $q(i) = i - N\,[i / N]$ corresponding to the proportion of the second coding state in a combined unit. So a function to the coding state for the i-th unit cell is derived as[3]

$$Code(i)=\begin{cases}\{([\frac{i}{N}]-1)/\frac{2\pi}{d\varphi_x}\}/\frac{d\varphi}{2\pi} & ,q<0.5\\ \{[\frac{i}{N}]/\frac{2\pi}{d\varphi}\}/\frac{d\varphi}{2\pi} & ,q\geq 0.5\end{cases} \qquad \text{(S4)}$$

where $[x]$ is an integer-valued function and $\{x\} = x - [x]$. Table S1 lists the fractional coding sequences (used in

the experiment) calculated by Eq. (S4).

Based on the fractional coding, we further predict the beam scanning range under different incident angles within 0°–30° (assuming a metasurface consisting of 64×64 elements with d = 200 μm, operating at 0.34 THz with the ideal 1-bit condition). Fig. S3 plots the angular variation of main and grating lobes in corresponding coding arrangements N (referring to Eq. (1) in the main text). Under normal and 15° incidences, the main-lobe angles are in good accordance with the predictions of Eq. (1). Increasing the incident angle, the main lobes and grating lobes will move together towards the positive angular range of 0°–60°, and the grating lobes nearly cover the negative angular range of -60°–0°. According to the calculation results, we have to avoid the occurrence of grating lobes in the beam-scanning view field for larger incident angles, which also can be inhibited by the numerical optimization of array codings.

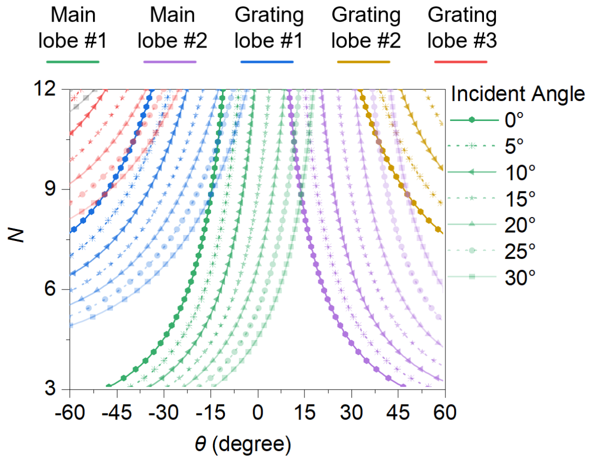

Fig. S3 Reflected main lobes and grating lobes under different incidence angles.

(2) Convolutional coding

The Fourier transform decomposes a time-domain signal into summing multiple frequency-domain signals for signal processing. According to the mapping relation between the coding metasurfaces and informatics, the phase distributions on the coding metasurfaces and corresponding beam patterns can be treated as time-domain and frequency-domain signals, respectively. Henceforth, more flexible beam manipulation can be realized by signal processing approaches. The convolution operation, as one of the interesting behaviors, can synthesize a beam pattern into another direction just as frequency spectrum shifting in signal processing. The Fourier transform of the convolution theorem is mathematically expressed as

$$f(t)\cdot g(t) \xleftrightarrow{\text{FFT}} f(\omega) * g(\omega) \quad \text{(S5)}$$

where the mapping relations meet[4]

$$\begin{cases} t \rightarrow \dfrac{\lambda}{Nd} \\ \omega \rightarrow \sin\theta \end{cases} \quad \text{(S6)}$$

The time-domain item is mapped to the phase (as exponential items), and the frequency-domain item is related to the beam directivity. Therefore, the multiplication of the phase of two coding matrixes is just equivalent to the modulus of their coding digits (for example, "0"+"0"="0", "0"+"1"="1", "1"+"0"="1", and "1"+"1"="0"). Fig. S4a illustrates the process of beam synthesis from the coding matrixes $\mathbf{C_a}$ with N=32 and $\mathbf{C_b}$ with N=5.5 to the convolutional coding matrix $\mathbf{C_{con}}$. Table S3 lists the convolutional coding sequences for multi-beam manipulations.

(3) Diffuse scattering based on GRS coding.

Golay-Rudin-Shapiro (GRS) polynomials are applied in many applications such as antenna arrays and communications due to their unique mathematical properties. There are two types of GRS polynomials with an entangled relation defined as

$$\begin{aligned} P_{\nu+1}(\xi) &= P_{\nu}(\xi) + \xi^{2^{\nu}} Q_{\nu}(\xi) \\ Q_{\nu+1}(\xi) &= P_{\nu}(\xi) - \xi^{2^{\nu}} Q_{\nu}(\xi) \end{aligned} \tag{S7}$$

where ξ is a complex-valued variable and the initial condition meets $P_0 = Q_0 = 1$. To simplify the analysis, we focus on the 1-bit cases with 1-D phase distributions and transform Eq. (S7) to the following form

$$\Lambda(\xi) = \sum_{x=1}^{X} e^{-j\varphi_x} e^{-jd_x(x-1)\sin\theta\cos\varphi} = \sum_{x=1}^{X} \Gamma_x \xi^x \tag{S8}$$

in which $\Gamma_x = 1$ for $\varphi_x = 0$ and $\Gamma_x = -1$ for $\varphi_x = \pi$, relating to 1-bit coding. The polynomial relations between Eq. (S7) and Eq. (S8) have been verified by Ref. [5]. Therefore, the absolutely continuous spectral property of the GRS polynomials can be used to engineer the coding sequences for diffuse scattering. The GRS coding sequences $\{C_x\}$ are determinately calculated from the recursive definitions in Eq. (S7). Simpler expressions are deduced as

$$S_0 = 1,\ S_{2x} = S_x,\ S_{2x+1} = (-1)^x S_x \tag{S9}$$

The P-type sequences are confirmed by

$$C_x = S_x, \quad x=0, \dots, X-1 \tag{S10}$$

And the Q-type sequences depend on

$$C_x = \begin{cases} S_x, & x=0, \dots, \dfrac{X}{2}-1 \\ -S_x, & x=\dfrac{X}{2}, \dots, X-1 \end{cases} \tag{S11}$$

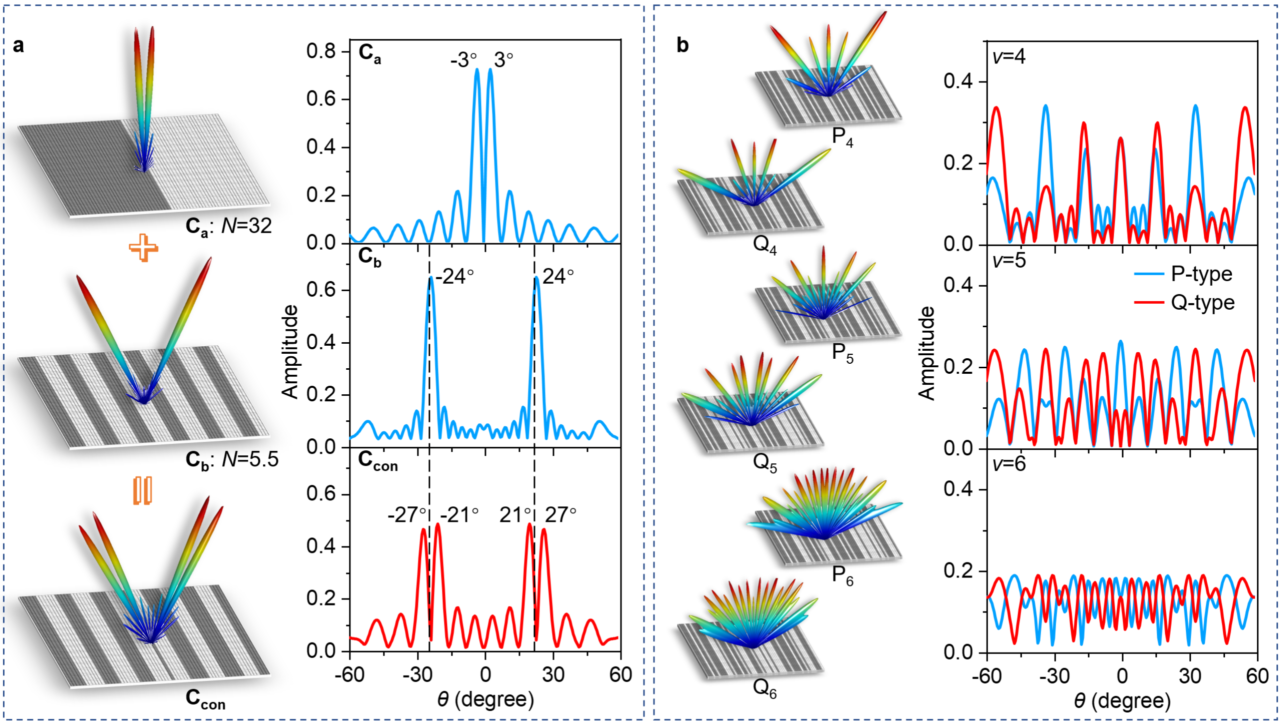

Fig. S4 Calculated beam patterns. **a** Based on convolutional coding. **b** Based on GRS coding.

The length of a sequence is defined as $L=2^{\nu}$ (ν=1, 2, …) to strictly relate to the GRS polynomials with spectral

flatness properties favorable for diffuse scattering. Fig. S4b shows the patterns of diffuse scattering by applying GRS coding with ν=4, 5, and 6 along the 1-D gradient direction. The maximum reflectance reduces from ~30% to ~20% with the sequence length increased from 2^4 to 2^6. Due to the inherent entangled relation, the P_ν- and Q_ν-type coding with the same length show comparable scattering ability, and the longer GRS sequence can present better spectral flatness properties to enhance diffuse scattering.

Note 4. Calculation of the beam gain

Antenna gain is defined as

$$G(\theta,\varphi) = 4\pi \frac{\text{radiation intensity}}{\text{total input power}} = 4\pi \frac{U(\theta,\varphi)}{P_{in}} \qquad \text{(S12)}$$

Which is related to the directivity by

$$G(\theta,\varphi) = e_r \frac{4\pi U(\theta,\varphi)}{P_{rad}} = e_r D(\theta,\varphi) \qquad \text{(S13)}$$

Where P_{rad} is total radiated power and e_r is radiation efficiency. For aperture antennas, the aperture area A determines the maximum directivity by

$$D = 4\pi \frac{A}{\lambda^2} \qquad \text{(S14)}$$

Here λ is the wavelength at the operating frequency. According to Eq. (S13) and Eq. (S14), the metasurface gain can be written as

$$G = e_{ap} \frac{4\pi A}{\lambda^2} \qquad \text{(S15)}$$

Where e_{ap} is the aperture efficiency and A is 13×13 mm^2 in this paper. The aperture efficiency is related to the illumination efficiency and reflection efficiency. In our experiments, we only focus on the performance of the metasurface, therefore the illumination efficiency determined by the feed horn antenna is normalized. The received power confirms the reflective efficiency in different directions.

Note 5. Tests of voltage-modulated characteristics

(1) DC responses of a single HEMT.

The single-HEMT DC characteristics are measured on the Agilent B1500A probe platform. As shown in Fig. S5, the drain-source current I_{DS} reaches the saturated state in the zone of V_{GS}≥5 V and is nearly cut off at the bias voltage V_{GS}=4 V, which shows a good electrically controlled performance of the single HEMT. Besides, we also find that the HEMT would be breakdown only when the bias voltage is greater than 10 V.

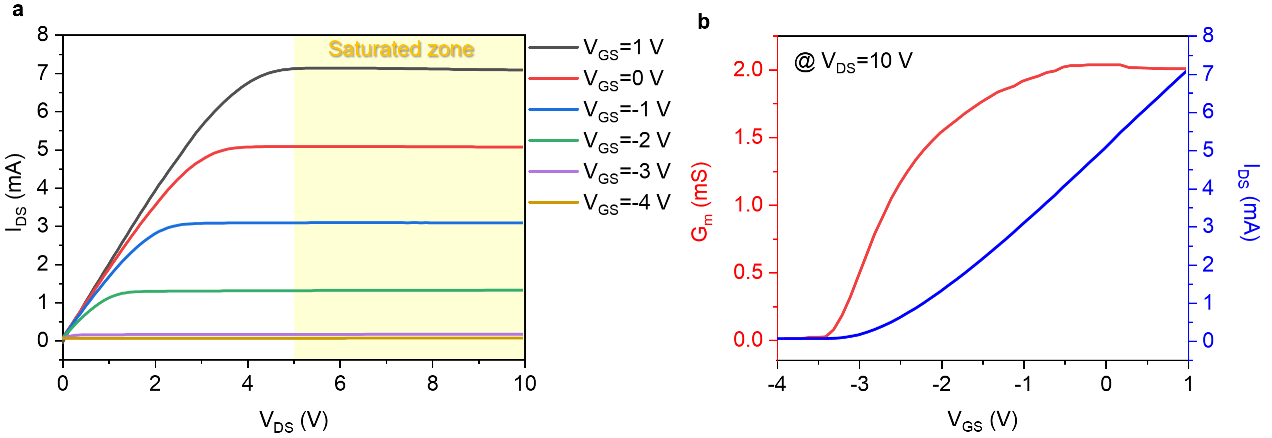

Fig. S5 The single meta-element DC characteristics. **a** The drain-source I-V curves with the gate-source voltages from 1

V to -4 V. **b** The transconductance at V_{DS}=10 V.

(2) Responses of amplitude and phase under bias voltages.

Fig. S6 displays the responses of amplitude and phase under different bias voltages so as to determine the voltage values corresponding to the "0" and "1" states by the 1-bit working condition (i.e., an on-off phase shift of 180° with the identical amplitude). As the carrier concentration decreases with increasing the bias voltage to -8 V, the on-state resonant frequency red-shifts, leading to a phase shift of 180° at 0.34 THz, shown as a black curve in Fig. S6b. The on-off amplitudes intersect at 0.34 THz on the amplitude curve shown in Fig. S6b, giving birth to the optimal 1-bit coding condition along with the phase modulation. Generally, the experimental amplitude/phase modulation trends and the central working frequency accord well with the simulation shown in Fig. 2. Acceptable deviations possibly come from fabrication errors and experiment errors, as have been discussed in the main text. Based on the above evidence, the "0" and "1" coding states thus correlate to 0 V and -8 V, respectively.

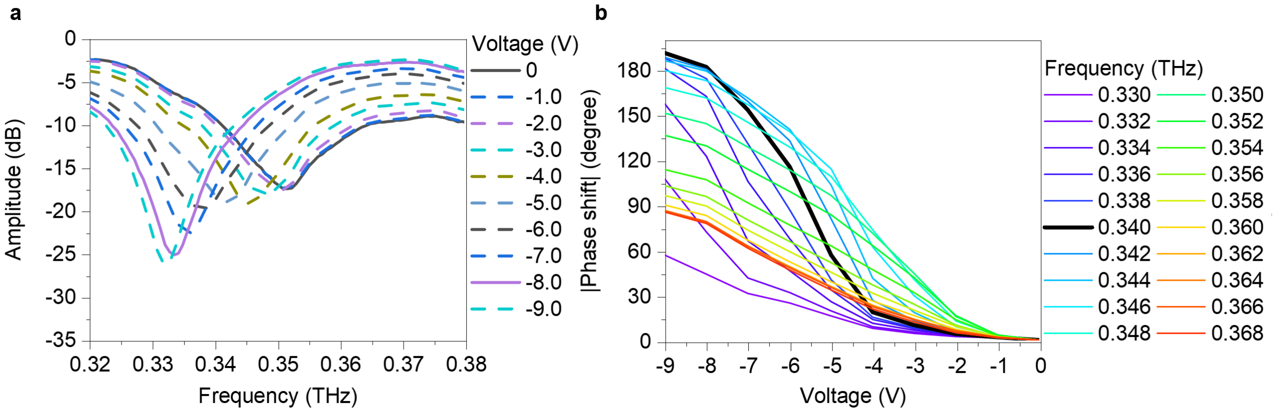

Fig. S6 The modulation characteristics corresponding to the different bias voltages. **a** The measured amplitude modulation. **b** The measured phase shifts related to the phase reference at 0 V.

Note 6. The response rate of the coding metasurface

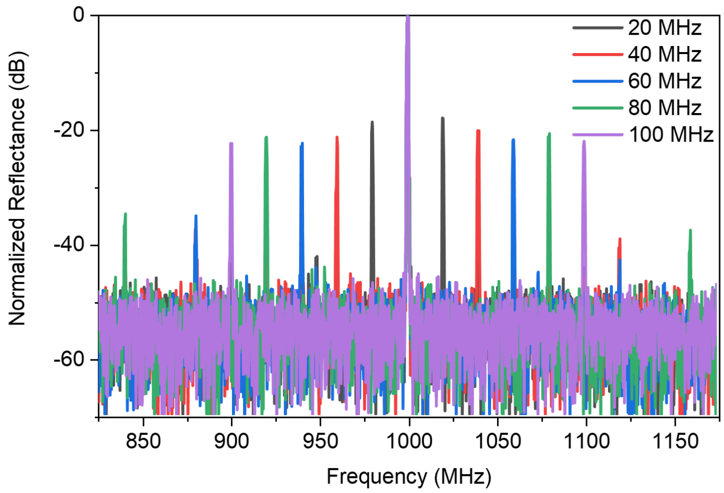

Fig. S7 Response rate of the coding metasurface.

We utilize this amplitude-modulation property of the metasurface to monitor the sideband signals by 0.34 THz carrier waves. The receiver accepts and demodulates signals by 1 GHz heterodyne reception at 0.339 THz. Square waves (on-off-key modulation) from the signal generator collectively control the metasurface. The receiver is

connected to the spectrometer to display demodulated signals, as shown in Fig. S7. The frequency shifts of available sideband signals correspond to the modulation speeds, implying response rates. Fig. S7 shows the available sideband signals from 20 MHz to 100 MHz, indicating that the response rate can reach 100 MHz.

Table S1 Fractional coding sequences

N	**Coding Sequences**	**Simulated Beam Angles**	**Measured Beam Angles**
3.75	…0 0 0 0 1 1 1 0 0 0 0 1 1 1 1…	57°	55°
4	…0 0 0 0 1 1 1 1…	53°	52°
4.25	…0 0 0 0 1 1 1 1 0 0 0 0 0 1 1 1 1…	50°	50°
4.5	…0 0 0 0 1 1 1 1 1…	48°	46°
4.75	…0 0 0 0 0 1 1 1 1 0 0 0 0 0 1 1 1 1 1…	46°	45°
5	…0 0 0 0 0 1 1 1 1 1…	44°	43°
5.5	…0 0 0 0 0 1 1 1 1 1 1…	41°	40°
6	…0 0 0 0 0 0 1 1 1 1 1 1…	38°	38°
6.5	…0 0 0 0 0 0 1 1 1 1 1 1 1…	36°	35°
7	…0 0 0 0 0 0 0 1 1 1 1 1 1 1…	35°	34°
8	…0 0 0 0 0 0 0 0 1 1 1 1 1 1 1 1…	32°	30°
9	…0 0 0 0 0 0 0 0 0 1 1 1 1 1 1 1 1 1…	30°	29°
10	…0 0 0 0 0 0 0 0 0 0 1 1 1 1 1 1 1 1 1 1…	28°	27°
11	…0 0 0 0 0 0 0 0 0 0 0 1 1 1 1 1 1 1 1 1 1 1…	27°	26°
12	…0 0 0 0 0 0 0 0 0 0 0 0 1 1 1 1 1 1 1 1 1 1 1 1…	26°	25°

Table S2 Dual-region coding sequences

N	**Coding Sequences**	**Simulated Beam Angles**	**Measured Beam Angles**
N=7/*N*=4	…0 0 0 0 0 0 0 1 1 1 1 1 1 1…/<br>…0 0 0 0 1 1 1 1…	35° / 53°	35° / 51°
N=8/*N*=5	…0 0 0 0 0 0 0 0 1 1 1 1 1 1 1 1…/<br>…0 0 0 0 0 1 1 1 1 1…	31° / 44°	31° / 42°
N=9/*N*=6	…0 0 0 0 0 0 0 0 0 1 1 1 1 1 1 1 1 1…/<br>…0 0 0 0 0 0 1 1 1 1 1 1…	29° / 39°	28° / 38°

Table S3 Convolutional coding sequences

N	**Coding Sequences**	**Simulated Beam Angles**	**Measured Beam Angles**
N=4.75**N*=32	…0 0 0 0 0 1 1 1 1 0 0 0 0 0 1 1 1 1 1…| …1 1 1 1 1 0 0 0 0 1 1 1 1 1 0 0 0 0 0…	42° / 50°	40° / 49°
N=5.5**N*=32	…0 0 0 0 0 1 1 1 1 1 1…<br>…1 1 1 1 1 0 0 0 0 0 0…	38° / 45°	36° / 44°
N=12**N*=32	…0 0 0 0 0 0 0 0 0 0 0 0 1 1 1 1 1 1 1 1 1 1 1 1…| …1 1 1 1 1 1 1 1 1 1 1 1 0 0 0 0 0 0 0 0 0 0 0 0…	23° / 29°	21° / 28°

Table S4 GRS coding sequences

**Types**	**Coding Sequences**
P4	…1 1 1 0 1 1 0 1 1 1 1 0 0 0 1 0…
Q4	…1 1 1 0 1 1 0 1 0 0 0 1 1 1 0 1…
P5	…1 1 1 0 1 1 0 1 1 1 1 0 0 0 1 0 1 1 1 0 1 1 0 1 0 0 0 1 1 1 0 1…
Q5	…1 1 1 0 1 1 0 1 1 1 1 0 0 0 1 0 0 0 0 1 0 0 1 0 1 1 1 0 0 0 1 0…
P6	1 1 1 0 1 1 0 1 1 1 1 0 0 0 1 0 1 1 1 0 1 1 0 1 0 0 0 1 1 1 0 1 1 1 1 0 1 1 0 1 1 1 1 0 0 0 1 0 0 0 0 1 0 0 1 0 1 1 1 0 0 0 1 0
Q6	1 1 1 0 1 1 0 1 1 1 1 0 0 0 1 0 1 1 1 0 1 1 0 1 0 0 0 1 1 1 0 1 0 0 0 1 0 0 1 0 0 0 0 1 1 1 0 1 1 1 1 0 1 1 0 1 0 0 0 1 1 1 0 1

Reference

1. Shrekenhamer, D. et al. High speed terahertz modulation from metamaterials with embedded high electron mobility transistors. Opt. Express, OE 19, 9968–9975 (2011).
2. Zhang, Y. et al. Large phase modulation of THz wave via an enhanced resonant active HEMT metasurface. Nanophotonics 8, 153–170 (2018).
3. Wang, L. et al. A fractional phase-coding strategy for terahertz beam patterning on digital metasurfaces. Opt. Express 28, 6395 (2020).
4. Liu, S. et al. Convolution Operations on Coding Metasurface to Reach Flexible and Continuous Controls of Terahertz Beams. Adv. Sci. 3, 1600156 (2016).
5. Moccia, M. et al. Coding Metasurfaces for Diffuse Scattering: Scaling Laws, Bounds, and Suboptimal Design. Advanced Optical Materials 5, 1700455 (2017).
